# A feature fusion network with spatial-temporal-enhanced strategy for the motor imagery of force intensity variation

**DOI:** 10.3389/fnins.2025.1591398

**Published:** 2025-06-20

**Authors:** Ankai Ying, Jinwang Lv, Junchen Huang, Tian Wang, Peixin Si, Jiyu Zhang, Guokun Zuo, Jialin Xu

**Affiliations:** ^1^Cixi Biomedical Research Institute, Wenzhou Medical University, Ningbo, Zhejiang, China; ^2^Ningbo Institute of Materials Technology and Engineering, Chinese Academy of Sciences, Ningbo, Zhejiang, China; ^3^Ningbo Cixi Institute of Biomedical Engineering, Ningbo, Zhejiang, China; ^4^Hangzhou RoboCT Technology Development Co., Ltd., Hangzhou, China; ^5^Ningbo College of Materials Engineering, University of Chinese Academy of Sciences, Beijing, China

**Keywords:** brain-computer interfaces, force intensity variation, spatial-temporal-enhanced strategy, motor imagery, deep learning

## Abstract

**Introduction:**

Motor imagery (MI)-based brain-computer interfaces (BCI) offers promising applications in rehabilitation. Traditional force-based MI-BCI paradigms generally require subjects to imagine constant force during static or dynamic state. It is challenging to meet the demands of dynamic interaction with force intensity variation in MI-BCI systems.

**Methods:**

To address this gap, we designed a novel MI paradigm inspired by daily life, where subjects imagined variations in force intensity during dynamic unilateral upper-limb movements. In a single trial, the subjects were required to complete one of three combinations of force intensity variations: large-to-small, large-to-medium, or medium-to-small. During the execution of this paradigm, electroencephalography (EEG) features exhibit dynamic coupling, with subtle variations in intensity, timing, frequency coverage, and spatial distribution, as the force intensity imagined by the subjects changed. To recognize these fine-grained features, we propose a feature fusion network with a spatial-temporal-enhanced strategy and an information reconstruction (FN-SSIR) algorithm. This model combines a multi-scale spatial-temporal convolution module with a spatial-temporal-enhanced strategy, a convolutional auto-encoder for information reconstruction, and a long short-term memory with self-attention, enabling the comprehensive extraction and fusion of EEG features across fine-grained time-frequency variations and dynamic spatial-temporal patterns.

**Results:**

The proposed FN-SSIR achieved a classification accuracy of 86.7% ± 6.6% on our force variation MI dataset, and 78.4% ± 13.0% on the BCI Competition IV 2a dataset.

**Discussion:**

These findings highlight the potential of this paradigm and algorithm for advancing MI-BCI systems in rehabilitation training based on dynamic force interactions.

## 1 Introduction

Motor imagery (MI) is a paradigm within the Brain-Computer Interface (BCI) framework, where users imagine body movements to activate specific brain regions (Brusini et al., 2021). When subjects perform MI, movement-related electrical activity emerges in the brain, giving rise to a range of neural responses (Yuan et al., 2010). Neural responses associated with motor intention generation reflect dynamic brain activity changes. Specifically, these responses serve as key electroencephalography (EEG) features for the analysis and interpretation of motor intentions, such as event-related desynchronization (ERD) and movement-related cortical potentials (MRCP) (Chaisaen et al., 2020; Savić et al., 2020). ERD is characterized by increased activity in relevant brain regions during motor imagery, which manifests as a decrease in EEG power within the alpha and beta frequency bands (Meirovitch et al., 2015). MRCP reflects the brain’s preparatory activity in the early stages before movement execution, characterized by gradual negative shifts in low-frequency EEG potentials (Zhang et al., 2024).

Motor imagery-based brain–computer interface (MI-BCI) systems translate users’ imagined movements into control signals, enabling users to control devices for tasks such as communication, assistance, and rehabilitation (Frisoli et al., 2012; Baniqued et al., 2021; Saha et al., 2021; Altaheri et al., 2023a). Such systems play a crucial role in stroke rehabilitation, with brain-controlled rehabilitation robots being one of its key applications.

The motor intentions of patients are recognized by MI-BCI systems in real time. MI-BCI systems control rehabilitation robots to execute corresponding actions, thereby facilitating the recovery of motor functions in patients. In MI-BCI systems, simple limb movement imagery tasks are commonly used, which typically instruct users to imagine movements with their left hand, right hand, or feet (Neo et al., 2021; Cristancho Cuervo et al., 2022; Rithwik et al., 2022; Ma et al., 2023). During the execution of simple limb motor imagery tasks, EEG signals primarily reflect activation in the sensorimotor areas, which are associated with movement planning and execution (Yi et al., 2013). However, such tasks typically lead only limited distinguishable features, as the recorded EEG signals mainly exhibit spatial pattern differences, such as stronger ERD in the right hemisphere during left-hand imagery and in the left hemisphere during right-hand imagery. These spatial patterns primarily reflect which limb is imagined as moving, rather than how the movement is imagined to occur, such as with a specific speed or force. Consequently, MI-BCI systems using such tasks can usually perform only basic directional commands such as “left” or “right.” However, effective rehabilitation requires more than the control of movement direction; it also involves the interaction of dynamic force between the robot and patient (Mohebbi, 2020). Given the variability in the patients’ muscle strength and motor abilities, it is essential for the robot to adapt its assistive force to meet individual needs. It is crucial to design a brain-controlled rehabilitation robot system based on motor imagery with dynamic force for the recovery of motor abilities (Liang et al., 2024). Accordingly, identifying the patients’ need for assistive force is crucial for MI-BCI systems. This facilitated the robot’s control of the force in the direction of movement.

In recent years, researchers have introduced force parameters in MI tasks. MI paradigms incorporating static force with a right-hand grip were designed, introducing multiple levels of force to analyze the variability in EEG features (Wang K. et al., 2017; Li et al., 2023; Yao et al., 2024). Differences in EEG features were found across the time, frequency, and spatial domains when subjects performed these paradigms. Traditional machine-learning algorithms have been designed and applied to classify these features for BCI control. Li et al. introduced two levels of force [5 and 40% maximum voluntary contraction (MVC)] and found differences in EEG in the frequency and spatial domains using power spectrum analysis and coherence analysis. Using a common spatial pattern (CSP) and support vector machine (SVM), they extracted and classified with an average classification accuracy of 81% (Li et al., 2023). Wang et al. used two levels of force (10 and 30% MVC). They analyzed the differences in ERD features in the frequency and spatial domains and the variations in MRCP in the temporal domain. ERD features were extracted using CSP, and the combined features of ERD and MRCP were classified by SVM, with an average classification accuracy of 78.3% (Wang K. et al., 2017). Bo et al. introduced four levels of force (15, 30, 45, and 60% MVC). Eight entropy metrics were utilized to classify the frequency-domain features, achieving an average classification accuracy of 70% (Yao et al., 2024). Distinct from MI paradigms with static force, Sheng et al. designed an MI paradigm with different force levels in a unilateral upper-limb dynamic state. They proposed a multi-scale temporal convolutional network with attention mechanism algorithm to recognize ERD features, with an average classification accuracy of 86.4% (Sheng et al., 2023). Nevertheless, existing paradigms maintain constant force within a single trial, limiting their applicability to natural interactions. In daily life scenarios, force tends to vary during movement, aligning more closely with natural interactions. This kind of natural interaction is crucial for brain-controlled rehabilitation robot systems, as it facilitates more realistic motor engagement.

By introducing force parameters into motor imagery tasks, the number of distinguishable feature classes in MI-BCI systems can be expanded. Different force levels within the same movement can elicit distinguishable EEG patterns that differ subtly in spectral, spatial, and temporal domains (Wang K. et al., 2017). However, constant force paradigms from previous studies are not well-suited for MI involving dynamic force in brain-controlled rehabilitation robot systems. Moreover, most of previous studies employed traditional machine learning approaches, with only a few exploring deep-learning (DL)-based algorithms. Machine learning methods require features to be selected manually with considerable expert knowledge, thereby limiting classification performance (Rashid et al., 2020; Altaheri et al., 2023b; Ahuja and Sethia, 2024; Khosravi et al., 2024). Due to the nonlinear and high-dimensional nature of EEG signals, machine learning methods may be less efficient and face challenges in capturing subtle difference in EEG patterns. In contrast, DL-based algorithms extract features directly from raw EEG signals, enabling end-to-end learning from input to classification without the need for manually extracting features (Dose et al., 2018; Roy et al., 2019; Kim et al., 2023; Hu et al., 2024). They are well-equipped to identify EEG patterns, but some limitations remain. Li et al. classified motor imagery EEG signals using a hybrid network combining a one-dimensional Convolutional Neural Network (1D CNN) and a Long Short-Term Memory Network (LSTM). The algorithm achieves an average accuracy of 87% for the test set (Li et al., 2022). However, the one-dimensional CNN-LSTM structure focuses only on feature extraction in a single direction, mainly along the temporal direction, which may ignore useful information from the spatial domain. Wang et al. designed a two-dimensional CNN-LSTM for recognizing motor intentions, which converted the EEG signals into time series segments, extracted the connectivity features between the different EEG electrodes in each segment using a two-dimensional CNN (2D CNN), and finally sent the feature vectors to an LSTM for training; the algorithm achieved an average accuracy of 93.3% on the test set (Wang et al., 2023). The above methods rely on single-scale kernels or single-branch structures, which limits their ability to capture subtle variations in EEG patterns across spectral, spatial, and temporal domains.

To address this challenge, recent studies have introduced multi-branch neural network architectures that are better suited for capturing subtle EEG pattern variations. Liu et al. employed a filter-bank structure combined with multi-scale temporal convolutions and spatial filtering to extract diverse time-frequency and spatial features (Liu et al., 2023). It achieved 79.17% accuracy on a four-class classification task. Qin et al. proposed a lightweight multi-branch network that integrates multiple attention modules to eliminate redundant frequency information and enhance the extraction of fine-grained spatial features (Qin et al., 2024). This method achieved superior classification accuracy compared to existing approaches on multiple MI datasets. Sheng et al. proposed a model for recognizing force MI tasks, which integrates an attention mechanism with a multi-scale CNN (Sheng et al., 2023). The architecture extracts features from time, frequency, and spatial domains with finer granularity. Its attention module highlights key components, enhancing sensitivity to subtle EEG differences. While these models improve time-frequency and spatial feature representation, EEG signals are still represented in a two-dimensional (2D) representation, with electrodes arranged on one axis and time steps on the other.

However, the 2D EEG representation inevitably leads to the loss of important spatial information, as it does not capture the true topological relationships among neighboring electrodes. Wang et al. proposed a multi-branch depthwise separable 2D convolutional network specifically designed to process 3D EEG representations (Wang X. et al., 2024). However, adapting 2D convolutions to 3D EEG data limits the model’s ability to fully capture spatial relationships among electrodes. Zhao et al. proposed a three-dimensional EEG representation combined with a multi-scale 3D convolutional neural network, achieving simultaneous extraction of time-frequency and spatial features while enhancing the spatial information (Zhao et al., 2019). It achieved superior accuracy on four-class MI classification, with enhanced inter-subject robustness. Furthermore, these methods primarily rely on ERD-based features, which restricts the diversity of learned representations. Beyond ERD, MRCP offer another dimension of motor-related EEG features. Given its low-frequency, time-locked nature, MRCP complement ERD by capturing different aspects of brain activity during motor imagery.

Therefore, we designed an MI paradigm for force intensity variations during dynamic movements of the unilateral upper- limb. In a single trial, subjects imagined variations in the force intensity of limb movements. We analyzed the time, time-frequency, and time-frequency-space domain features of the EEG signals induced by this paradigm. The ERD features of the EEG signals exhibit dynamic time-based changes in spatial distribution and frequency range, which influenced by variations in force intensity. To address this complexity, we introduced a multi-scale spatial-temporal convolutional network module that learns the spatial-temporal-enhanced features of EEG signals using three-dimensional convolutions and electrode rearrangement. The EEG noise of specific electrodes was reduced by a convolutional auto-encoder module, which effectively reconstructed low-frequency information (MRCP-based features) and enriched the recognizable features of the EEG signals. Finally, to address individual differences among subjects during the MI tasks, an LSTM combined with a self-attention mechanism was adopted to adaptively fuse the extracted features. This integrated architecture enhances the model’s capacity to capture fine-grained variations in EEG patterns, ultimately improving single-trial classification performance and supporting the development of MI-BCI systems capable of dynamic force interaction.

## 2 Materials

### 2.1 Dataset 1

#### 2.2.1 Subjects

An advertisement seeking participants was posted at the Ningbo Institute of Material Technology and Engineering, outlining the basic details of the study, including the purpose, experimental procedures involving motor imagery tasks, and participation requirements. Participants were informed that they would receive a compensation of 50 CNY per hour as a reimbursement for their time and effort, with a minimum participation duration of 3 h.

Twenty subjects participated in the experiment. None of the participants had a history of neurological or movement disorders. None of them had prior experience with EEG or BCI. During the preprocessing, data from three participants were excluded due to poor EEG data quality (e.g., severe drift and numerous muscle artifacts). Therefore, EEG data from 15 participants (8 males and 7 females, right-handed, mean age 23.87 ± 1.85 years) were retained for analysis. Before the experiment, all subjects were informed of the experimental procedures and signed an informed consent form for the experimental study. In addition, a 1-week training session was arranged to familiarize the participants with the experimental procedures (Zhang et al., 2011). All experiments were conducted in line with the principles of the Declaration of Helsinki, and the study was approved by the Ethic Committee of Ningbo Institute of Material Technology and Engineering, Chinese Academy of Sciences (protocol number:2024021800005).

#### 2.2.2 Experimental procedure

The entire experiment was conducted in an electromagnetically shielded room to block electromagnetic signals and background noise interference, ensuring the accuracy of the experimental results. The subjects sat in a comfortable armchair in front of a computer screen, with their hands resting naturally on the armrests. Before EEG recording, all subjects completed a brief training session to become familiar with the experimental procedure and motor imagery tasks. They were instructed on how to perform MI and practiced several trials using the same visual cues as in the formal experiment. Real-time guidance was provided by the experimenter to ensure that all subjects accurately understood the task. Training continued until subjects reported confidence in performing the tasks as instructed.

Based on the action of pouring water in human daily life, we designed an MI paradigm in which subjects imagined variations in force intensity during dynamic movements of the unilateral upper-limb in a single trial. In this experiment, we selected the right upper-limb as the unilateral limb. Three combinations of force intensity variations were introduced: large-to-medium force, large-to-small force, and medium-to-small force. This enhanced the variety of categories of MI thinking. The MI scenarios correspond to pouring water from a thermal pitcher into a glass teapot and then from the glass teapot into a cup (large-to-medium force, LMF), pouring water from a thermal pitcher into a cup, lifting the cup (large-to-small force, LSF), and pouring water from a glass teapot into a cup and then lifting the cup (medium-to-small force, MSF). The levels of force intensity in this paradigm were designed based on the amount of water in the lifted container, making it easier for the subjects to imagine.

**FIGURE 1:**
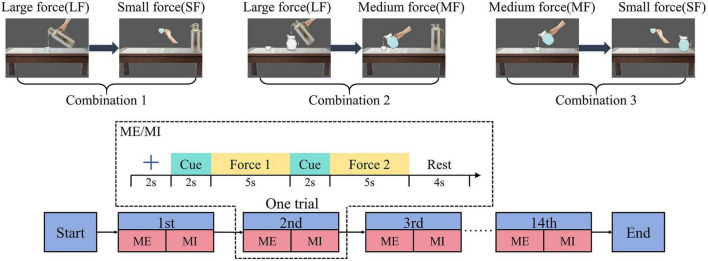
The experimental procedure diagram indicates that, in a single trial, each participant needs to complete MI after ME. Three combinations of force intensity variations (large to small force, large to medium force, medium to small force) corresponds to three scenarios of pouring water.

Each subject was required to complete nine rounds of the experiments, with each round consisting of 14 individual trials. Hence, there were a total of 126 trials (42 trials each for the LMF, LSF, and MSF, respectively). The label organization consists of three class labels corresponding to the three MI tasks: LMF (class 0), LSF (class 1), and MSF (class 2). Each trial consisted of two task types: motor execution (ME) and MI. The purpose of this design involving ME followed by MI in the same trial was to enhance participants’ recall of muscle activation sensations after the ME task (Fukumoto et al., 2024). This helps them to perform accurate force imagery. The subjects were given a 5–10 min rest after each round of the experiment. The experimental flow is shown in Figure 1. The MI task was divided into six periods with a total time of 20 s. The first period was the preparation period, where a fixed cross appeared in the center of the computer screen for 2 s, with the subjects remaining relaxed. The second and fourth time periods were the force cue periods, in which the textual cue appeared for 2 s and the subjects remained relaxed without movement. The third and fifth periods were the MI periods, in which the textual cues disappeared from the screen, and the black screen lasted for 5 s. The subject then executed the MI. The sixth period was the rest period, in which the text “rest” appeared on the screen and lasted for 4 s, and the subjects remained relaxed. The ME process is similar to that of the MI process. The difference is that during the ME phase, the corresponding animations are presented on the screen as cues (Wang L. et al., 2017).

#### 2.2.3 Data acquisition and preprocessing

EEG data were recorded using a Neuroscan digital EEG system with a 64-channel Ag/AgCl electrode cap (Neuroscan Quik-Cap), following the international 10–20 system. Additional monopolar electrodes were placed at the left and right mastoids (M1, M2), while two bipolar electrode pairs recorded vertical and horizontal EOG (VEOG/HEOG) to detect eye movements and blinks. The reference electrode (REF) was positioned at the top of the head, and the ground electrode (GND) at the forehead (AFz). Signals were amplified using a SynAmps2 amplifier with a gain setting of 2010, a 0.05–200 Hz bandpass filter, and a 50 Hz notch filter to remove industrial frequency interference. The sampling rate was 1,000 Hz, and electrode impedance was maintained below 10 kΩ to ensure signal quality.

EEG data preprocessing was performed using the EEGLAB toolbox (v2023.0) (Delorme and Makeig, 2004). First, a 0.1–30 Hz Finite impulse response (FIR) bandpass filter was applied to remove low-frequency drift and high-frequency noise. Then, Common Average Reference (CAR) rereferencing was performed to reduce common noise interference. Horizontal (HEOG) and vertical (VEOG) electrooculographic artifacts were removed to minimize the impact of eye movements. The data were then downsampled to 100 Hz. Finally, a 12-second EEG segment was extracted from each trial, encompassing the first MI phase (5 s), the second preparation phase (2 s), and the second MI phase (5 s). Baseline correction was applied using 1 second of data preceding the first MI phase.

### 2.2 Dataset 2

The BCI Competition IV 2a dataset is used in this paper, a publicly available EEG dataset designed for MI classification (Tangermann et al., 2012). It contains EEG recordings from nine healthy subjects performing four MI tasks: imagining movements of the left hand, right hand, both feet, and tongue. EEG signals were recorded using 22 Ag/AgCl electrodes positioned according to the 10-20 system, with three additional electrooculogram (EOG) channels for eye movement monitoring. The signals were sampled at 250 Hz, bandpass-filtered between 0.5 and 100 Hz, and notch-filtered at 50 Hz. Each subject completed two sessions on different days, with each session consisting of six runs and each run containing 48 trials (in each session, each class had 72 trials). The label organization consists of four class labels corresponding to the four MI tasks: left hand (label 0), right hand (label 1), feet (label 2), and tongue (label 3). The first session serves as training data, while the second is used for testing, making this dataset a standard benchmark for MI-BCI research.

## 3 Methods

### 3.1 Event-related spectral perturbation

The Event-Related Spectral Perturbation (ERSP) is commonly used to observe the event-related power variations of the EEG signals in time and frequency domain (Yi et al., 2013). These power variations correspond to the ERD/ERS phenomena generated in the brain when subjects execute motor imagery (Wang Z. et al., 2024). The formula is as follows:


$$ERSP\left(f,t\right)=\frac{1}{n}\sum_{k=1}^{n}\left(F_{k}{\left(f,t\right)}^{2}\right)$$

Where *n* is the number of trails, and *F*_*k*_(*f*,*t*) is the power spectral density of *k*th trial at frequency *f* and time *t*. And in order to quantify the ERD patterns, we take the start moment of MI task (at the 4th second in Figure 1) as the time 0 s and –2 to 0 s as the baseline period. Average ERSP values were calculated with frequency ranging from 8 to 30 Hz and time ranging from –2 to 12 s. The contralateral motor control principle indicates that right-hand activity corresponds to activation in the left motor cortex (Pfurtscheller and Neuper, 2001). As our MI tasks focused on the right hand, we selected the C3 electrode positioned over the left motor cortex for analysis. Time-frequency maps from this electrode were subsequently examined.

To further analyze ERD/ERS phenomena in the spatial domain when performing LMF, LSF, and MSF tasks, the brain topographic map was drawn based on the average ERSP values from 0 to 12 s in alpha frequency band (8-13 Hz) and beta frequency band (13-30 Hz). In order to reveal the patterns of power changes with time and frequency in specific cortical regions during different tasks, ERSP wave maps is drawn based on average ERSP values from 0 to12 s in the electrode C3.

Through the above time-frequency and spatial analysis methods, the features of the EEG signals collected in this experiment can effectively substantiate the validity of our paradigm. This approach ensures that our paradigm accurately induces brain activity and provides solid signal support for subsequent algorithm design.

### 3.2 MI-EEG feature recognition algorithm

**FIGURE 2:**
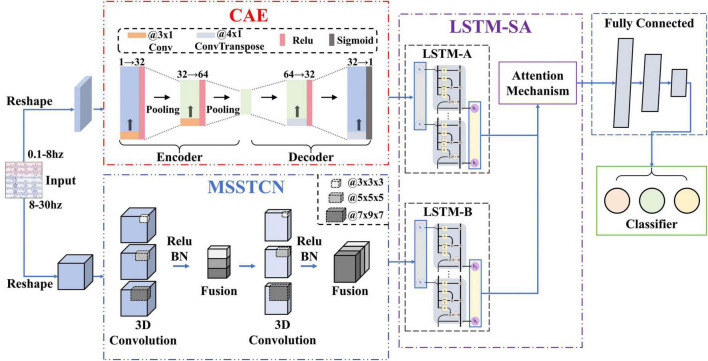
Network structure diagram. The feature fusion network with spatial-temporal-enhanced strategy and information reconstruction (FN-SSIR) consists of three major modules: the MSSTCN module, the CAE module, and the LSTM-SA module.

We proposed an algorithm to recognize the features of MI-EEG signals, which is a feature fusion network with spatial-temporal-enhanced strategy and information reconstruction (FN-SSIR). The network structure diagram is shown in Figure 2, and the network structure parameters are shown in Table 1. A multi-scale spatial-temporal convolutional module (MSSTCN) with spatial-temporal-enhanced strategy is employed to dynamically capture local details and global trends of EEG features across time, frequency, and spatial dimensions. Compact and meaningful low-frequency information is effectively extracted and reconstructed by a convolutional auto-encoder module (CAE). Then these features are weighted and fused through an LSTM with self-attention module (LSTM-SA), and classification was completed by a fully connected layer.

**TABLE 1:** The network structure parameters.

Module	Type	Layer	Parameter	Activation
	Input	Input		
	Reshape	Reshape1		
	Reshape	Reshape2		
MSSTCN	Convolution	Conv3d1	(3,3,3)	ReLU
	Batch normalization	BN1		
	Convolution	Conv3d2	(5,5,5)	ReLU
	Batch normalization	BN2		
	Convolution	Conv3d3	(7,9,7)	ReLU
	Batch normalization	BN3		
	Average pooling	Apool1	(3,3,5)	
	Convolution	Conv3d4	(3,3,3)	ReLU
	Batch normalization	BN4		
	Convolution	Conv3d5	(5,5,5)	ReLU
	Batch normalization	BN5		
	Convolution	Conv3d6	(7,9,7)	ReLU
	Batch normalization	BN6		
	Average pooling	Apool2	(3,3,10)	
	Droput	Drop	p = 0.5	
CAE	Convolution	Conv2d1	(1,3)	ReLU
	Max pooling	Mpool1	(1,2)	
	Convolution	Conv2d2	(1,3)	ReLU
	Max pooling	Mpool2	(1,2)	
	Transposed convolution	ConvTranspose1	(1,4)	ReLU
	Transposed convolution	ConvTranspose2	(1,4)	Sigmoid
LSTM-SA	LSTM	LSTMA	40	
	LSTM	LSTMB	80	
	Self-attention	Self-attention	120	
	Flatten	Flatten		
Parameter *p* is the dropout rate.				

#### 3.2.1 MSSTCN module

Through the time-frequency analysis based on ERSP, we discovered that with the reduction of force intensity in MI tasks, the ERD phenomenon was gradually weakened, especially in duration and frequency range. Additionally, brain topographic maps for alpha frequency band showed that contralateral sensorimotor areas of the brain were activated when the subjects performed MI tasks. As it was a motor imagery of the right upper-limb, the activated areas were concentrated around the C3 electrode. The degree and extent of activation were also positively correlated with the variations of force intensity. The features of EEG signals reflected by the ERD were dynamic time-based change and interrelated in multiple dimensions. Although these features can be extracted by the single scale 2D CNN (Fu et al., 2024), it ignores the spatial relationship between electrodes. As a result, the ability to extract high-dimensional features is limited.

**FIGURE 3:**
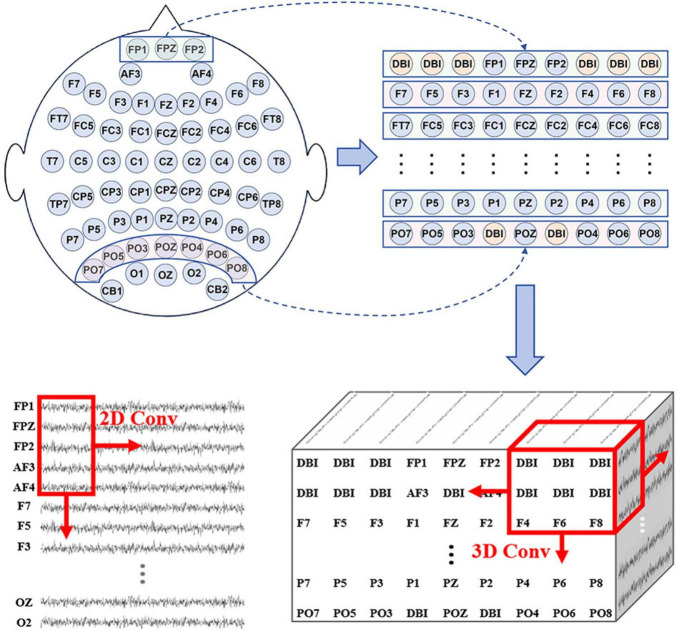
According to the electrode distribution of the brain topographic maps, the one-dimensional time series on N electrodes were arranged to form the electrode plane, (distance-based interpolation, DBI).

Consequently, we use the MSSTCN module with spatial-temporal-enhanced strategy as the first part of the FN-SSIR model. The module consists of two multi-scale spatial-temporal convolution units. The parameters of the module structure are shown in Table 1. The MSSTCN module can capture both local details and global trends of EEG signals by combining small and large convolutional kernels. Subtle differences in time-frequency features near local electrodes can be detected by small convolutional kernels. Whereas global dynamic change features based on ERD across a broader range can be captured by large convolutional kernels. As illustrated in Figure 3, the 2D convolutional kernel slides along only two directions (time series direction, one-dimensional electrode direction). Referring to the electrode distribution of the brain topographic maps, the one-dimensional time series on *N* electrodes were arranged to form the electrode plane, according to the two-dimensional arrangement of electrodes. We used the distance-based interpolation method to fill vacant electrode in this plane (Courellis et al., 2016). The 3D convolution (3D Conv) can slide in three directions (time series direction, height direction and width direction of the plane), which enhances the ability of capturing spatial-temporal information. Define the input of this module to be *X* ∈ *R*^1×*H*×*W*×*T*^ with the following formula:


$$\begin{array}{c} X_{i}^{1}=C\,o\,n\,v_{i}\,\left(X\right),i=1\,\cdots \,3 \end{array}$$

$$\begin{array}{c} X_{h\,i\,d\,e}=H\,\left(C\,a\,t\,\left(X_{1}^{1},X_{2}^{1},X_{3}^{1}\right)\right) \end{array}$$

$$\begin{array}{c} X_{i}^{2}=C\,o\,n\,v_{i}\,\left(X_{h\,i\,d\,e}\right),i=1\,\cdots \,3 \end{array}$$

$$\begin{array}{c} X_{o\,u\,t}=H\,\left(C\,a\,t\,\left(X_{1}^{2},X_{2}^{2},X_{3}^{2}\right)\right) \end{array}$$

where *H* is the height of the electrode plane, W is the width of the electrode plane, *T* is the number of time points of the EEG data, and *H*(⋅) is a composite layer consisting of four continuous units as we defined. The four continuous processing units are the batch normalization (BN) layer, the rectified linear unit (Relu) (Ioffe and Szegedy, 2015), the average pooling layer, and the dropout layer.

#### 3.2.2 CAE module

We found that there was a significant difference beginning at 0.5–1.5 s after the onset of MI tasks, through analysis of the temporal waveform. The corresponding waveform gets more negative as the force intensity gets larger. Because MRCP is a low frequency neural oscillations with a low signal-to-noise ratio, the waveform features in a single trial are difficult to be effectively extracted (Xu et al., 2014). Therefore, we used a convolutional auto-encoder module to reconstruct information for noise reduction of EEG signals in specific electrodes (FC3, FCZ, FC3, C3, CZ, C4). In the encoder, key temporal information, including onset time and duration related to motor imagery is gradually extracted by multiple convolutional layers. The pooling layer reduces the dimensionality to further extract information. This information is reconstructed through transposed convolution (ConvTranspose) in the decoder. We define the input as *X*_*m*_ ∈ *R*^*C*×*T*^. The formula is as follows:


$$\begin{array}{c} X_{e\,n\,c\,o\,d\,e\,r}=K\,\left(C\,o\,n\,v_{2}\,\left(K\,\left(C\,o\,n\,v_{1}\,\left(X_{m}\right)\right)\right)\right) \end{array}$$

$$\begin{array}{c} X_{d\,e\,c\,o\,d\,e\,r}=C\,o\,n\,v\,T_{2}\,\left(R\,e\,L\,U\,\left(C\,o\,n\,v\,T_{1}\,\left(X_{e\,n\,c\,o\,d\,e\,r}\right)\right)\right) \end{array}$$

where *C* is the electrode, *T* is the number of time points of the EEG data, and *K* is a composite layer consisting of two continuous units as we defined. The two continuous units are the rectified linear unit (Relu), and the max pooling layer.

#### 3.2.3 LSTM-SA module

In the brain topographic maps of alpha frequency band across segmented time periods, there are significant disparities in the activation of contralateral sensorimotor areas among different subjects when they imagined the variations in the force intensity of limb movements. This variation may be attributed to individual differences in the onset time and duration of motor imagery. Therefore, an LSTM module combined with a self-attention mechanism is adopted (Vennerød et al., 2021; Vaswani et al., 2023). The module dynamically focuses on various segments of the input sequence at each time step and adjust its attention adaptively. The output features of the MSSTCN module and the CAE module are weighted according to their importance along the temporal dimension. Combining the memory capability of LSTM and the global information processing capacity of self-attention, it can capture and utilize long-range dependency information more effectively. The formula is as follows:


$$\begin{array}{c} X_{o\,u\,t}=F\,l\,a\,t\,t\,e\,n\,\left(S\,e\,l\,f\,a\,t\,t\,\left(L\,S\,T\,M\,\left(X_{i}\right)\right)\right),i=1,2 \end{array}$$

where *X*_*1*_ and *X*_*2*_ are the outputs of the multi-scale spatial-temporal convolutional module and the convolutional auto-encoder module, respectively.

The self-attention mechanism (Vaswani et al., 2023) consists of three key components: Query, Key, and Value. We take the long-term dependence of the EEG features captured by LSTM as the input matrix. Query matrix *Q_i_*, Key matrix *K_i_*, and Value matrix *V_i_* are generated by different linear transformations of the input matrix. The pairwise similarity across each Query matrix and all Key matrices is computed by the dot product between the *Q_i_* and *K_i_*. Then the pairwise similarity deflated by the scaling factor *d*. The weight scores of the values are obtained by using the *Softmax* function. The formula is as follows:


$$\begin{array}{c} A\,t\,t\,e\,n\,t\,i\,o\,n\,\left(Q,K,V\right)=S\,o\,f\,t\,m\,a\,x\,\left(\frac{Q_{i}\,K_{i}^{T}}{\sqrt{d}}\right)\,V_{i} \end{array}$$

### 3.3 Experiments

#### 3.3.1 Baseline methods

To validate the advantages of the FN-SSIR, we select several excellent algorithms from current EEG features recognition researches. These selected algorithms serve as baseline algorithms for comparison with the FN-SSIR. These baseline algorithms are described as follows:

OVR-CSP+SVM (Cortes and Vapnik, 1995; Mu et al., 2023): CSP distinguishes different EEG activity patterns in motor imagery tasks by extracting the spatial features of EEG signals, and combined with SVM classifiers, which is widely used in BCI applications.

MSTCN-AM (Sheng et al., 2023): It is a model for the forceful motion imagery recognition that combines an attention mechanism and a multi-scale convolutional network to extract the time-frequency-space domain features and the attention module focuses the information of the time-frequency-space domain features.

EEGNet (Lawhern et al., 2018): A lightweight convolutional neural network, designed for processing EEG data, is able to efficiently extract spatial-temporal features of EEG signals. It achieves efficient recognition of different EEG patterns by combining deep and separable convolutional structures.

2D CNN-LSTM (Wang et al., 2023): Combining 2D CNN and LSTM, the original EEG data is segmented into time series segments, 2D CNN extracts the EEG signal channel connectivity features of each segment from two dimensions, and LSTM is utilized to effectively capture the long-term dependencies in the time-series data.

FBMSNet (Liu et al., 2023): an end-to-end filter-bank multi-scale convolutional neural network for MI classification. It extracts multi-scale spectral-temporal features and reduces volume conduction through spatial filtering.

BrainGirdNet (Wang X. et al., 2024): a CNN framework that integrates two intersecting depthwise CNN branches with 3D EEG data to decode multi-class MI task. The dual-branch structure enables complementary learning of spatial-temporal and spectral-temporal features, enhancing decoding performance across multiple domains.

### 3.3.2 Training procedure

All experiments were conducted in a high-performance computing environment with an Intel Core i9-12900KF @3.20GHz CPU, NVIDIA GeForce RTX 3090 GPU, and 80GB RAM. The models were implemented using the PyTorch deep learning framework and trained in a supervised setting.

For Dataset 1, the training of deep learning models was performed for 200 epochs using the Adam optimizer (learning rate = 0.001, batch size = 7), with the cross-entropy loss function guiding parameter updates. For Dataset 2, all deep learning models were trained for 400 epochs with a learning rate of 0.001 and a batch size of 36. The models were optimized using the Adam optimizer with cross-entropy loss. For the OVRCSP-SVM, we set the CSP component number (n_components) to 4, and the SVM was configured with a Gaussian radial basis function (RBF) kernel.

Regarding dataset partitioning, we adopted two different strategies according to dataset types. For Dataset1 (our private dataset), we performed four-fold cross-validation repeated five times. The final performance was reported as the highest average accuracy across the five runs. For Dataset2 (BCI Competition IV 2a), the first session of each subject was used for training, and the second session for testing, in accordance with the official competition protocol.

## 4 Results

### 4.1 MI-EEG feature analysis results

**FIGURE 4:**
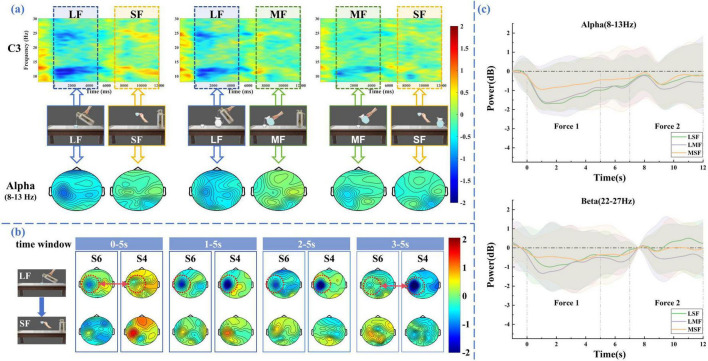
MI-EEG feature analysis results. **(a)** The average time-frequency maps of C3 electrode and the brain topographic maps in the alpha frequency band (8–13 Hz). A power decrease (Blue) indicates the ERD phenomenon when subjects performing MI task. **(b)** The brain topographic maps (8–13 Hz) within the four time windows (0–5, 1–5, 2–5, and 3–5 s) during the motor imagery phase. **(c)** ERSP curves in alpha band and beta band. The curves show the changes of power (in dB) with time (in second).

To investigate the time-frequency characteristics of MI-EEG when subjects imagined the variations in the force intensity of limb movements, we performed time-frequency analysis using ERSP. A 1-second pre-stimulus interval was used as the baseline for correction. Figure 4a shows the average time-frequency maps at electrode C3 across all subjects when they performed three types of motor imagery tasks: large-to-medium, large-to-small, and medium-to-small. Clear ERD responses were observed in both the alpha (8–13 Hz) and beta (13–30 Hz) bands after the onset of the MI period. Notably, during the MI tasks, as the force intensity imagined by the subjects decreased, the ERD became shorter in duration, particularly in the 8–13 Hz and 22–27 Hz bands. To further quantify the ERD differences, we computed the average ERSP power at the C3 electrode within two frequency bands: 8–13 Hz (alpha) and 22–27 Hz (beta). The corresponding time-resolved power curves are shown in Figure 4c. The results indicate that, the ERD magnitude in both bands progressively decreased with the reduction in force intensity during the execution of MI tasks.

To further examine the spatial distribution, we averaged the ERSP values in the alpha band (8–13 Hz) and plotted the corresponding scalp topographies. These maps revealed contralateral activation patterns in the sensorimotor cortex, with the extent of activation decreasing as force intensity imagined by the subjects declined. These results further confirm that different force levels can modulate cortical activation strength during MI, as previously reported (Wang K. et al., 2017).

To further illustrate inter-subject variability, we plotted the alpha-band (8–13 Hz) topographic maps of two representative subjects (S4 and S6) across four time windows: 0–5s, 1–5 s, 2–5 s, and 3–5 s, as shown in Figure 4b. Clear differences in the onset and persistence of ERD can be observed between subjects. For example, in the 0–5 s window, subject S6 exhibits a pronounced ERD over the contralateral sensorimotor cortex, while subject S4 shows only a weak response. In contrast, during the 1–5 s and 2–5 s windows, both subjects display clear ERD patterns. However, in the 3–5 s window, ERD in S6 diminishes, whereas S4 maintains a strong ERD response. These results suggest that the temporal profile of ERD varies across individuals, which is reflected in the variability of the onset and duration of cortical activation.

**FIGURE 5:**
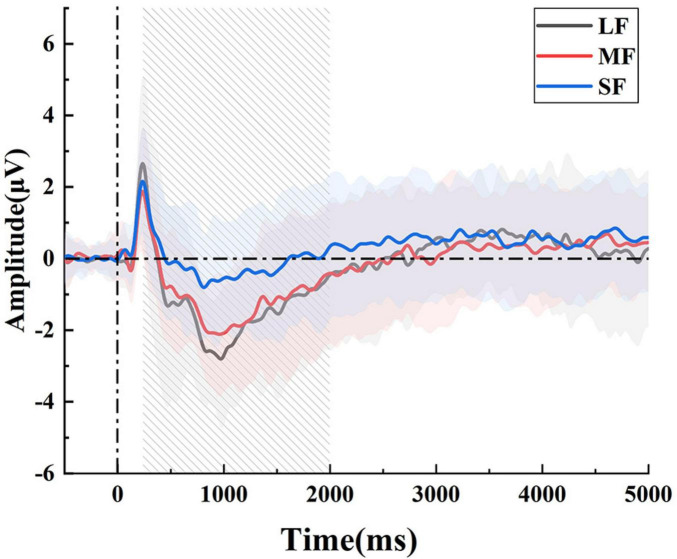
The average MRCP waveforms in the FC3 electrode. There were significant differences, particularly at the 1st second after the onset.

Since MRCP are low-frequency and low-amplitude signals that are easily contaminated by noise, we performed trial averaging to improve the signal quality. Based on the averaged signals, we plotted the MRCP waveforms of the FC3 electrode for all subjects during the early stage of MI tasks. As shown in Figure 5, a noticeable negative shift began approximately 0.5 s after the initiation of the MI task and peaked around 1 second. This negative deflection reflects the activation of the motor cortex associated with motor preparation. Notably, imagery tasks with larger force consistently induced larger negative amplitudes compared to those with smaller force. This pattern suggests that MRCP not only reflect the initiation of motor intentions but are also modulated by the intensity of motor effort imagined by the subjects.

### 4.2 Recognition results

We designed and optimized the model based on the characteristics of Dataset1 (a force-varying MI-EEG dataset collected through our own experiments). On this dataset, we conducted comprehensive evaluations, including baseline comparisons, feature visualization, ablation studies, and cross-subject generalization analysis, to thoroughly assess the model’s performance, interpretability, and robustness.

**FIGURE 6:**
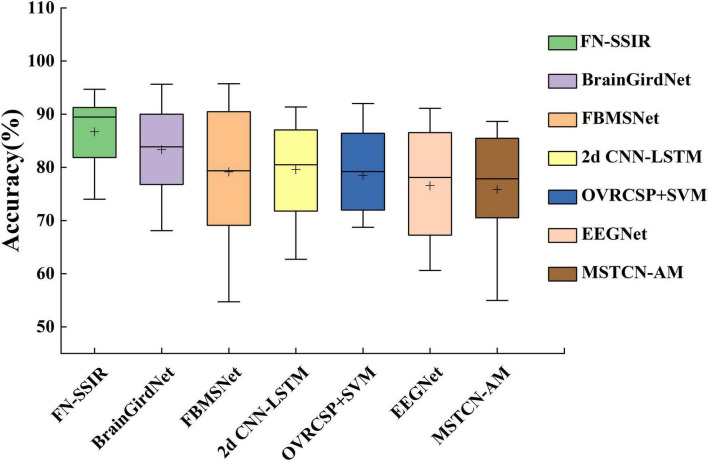
Box plot of classification accuracy of the FN-SSIR and other baseline algorithms. The box plot shows the comparison of classification accuracy results in Table 2 (“+” represents the average value).

A 4-fold cross-validation strategy was adopted to assess model performance. We calculated the classification accuracy and standard deviation for each model in Table 2, and summarized the results using box plots (Figure 6). FN-SSIR achieves the highest average accuracy of 86.7% and the lowest standard deviation of 6.6%, outperforming other baseline algorithms.

**TABLE 2:** Classification accuracy (%) comparison for different algorithms on dataset 1.

	FN-SSIR	BrainGird Net	FBMS Net	2d CNN- LSTM	OVR-CSP +SVM	EEGNet	MSTCN- AM
S1	92.4	90.3	88.1	80.5	86.4	86.6	85.5
S2	89.4	89.5	88.1	82.9	80.9	87.3	88.7
S3	83.7	83.2	90.5	85.8	79.2	91.1	86.6
S4	91.3	87.4	64.7	82.2	88.0	77.8	76.2
S5	85.8	70.3	69.1	68.1	68.7	67.3	63.5
S6	90.0	83.1	74.4	79.7	72.0	79.4	80.6
S7	94.7	95.6	79.4	71.8	72.1	78.8	78.5
S8	90.7	92.3	92.0	90.6	80.8	89.7	86.0
S9	74.1	68.1	59.9	62.7	69.6	63.9	70.8
S10	81.9	83.9	77.5	87.0	71.1	80.1	77.6
S11	93.3	90.0	93.7	91.3	76.0	78.1	79.2
S12	74.0	73.2	64.6	72.2	80.0	60.6	55.0
S13	89.5	85.0	91.8	88.2	92.0	74.7	77.9
S14	79.9	76.8	85.3	79.9	72.8	61.9	60.7
S15	89.6	81.2	76.3	70.8	87.1	71.2	70.5
Mean ± Std	86.7 ± 6.6	84.6 ± 7.8*	79.6 ± 8.7*	78.4 ± 7.4**	76.6 ± 9.9**	75.8 ± 9.9***	74.2 ± 8.6***

Stars denote the statistically significant difference between two algorithms (where *denotes *p* < 0.05, ^**^Denotes *p* < 0.01, and ^***^denotes *p* < 0.001).

A paired-sample t-test was performed to evaluate whether the differences in accuracy between our model and the baseline methods were statistically significant. The null hypothesis (H_0_) was that there was no significant difference in classification accuracy between our method and each baseline method, while the alternative hypothesis (H_1_) was that our method achieved a significantly different accuracy. Before conducting the t-test, we verified that the differences in accuracy between paired samples were approximately normally distributed, satisfying the normality assumption required for a paired-sample t-test. The outcomes of this statistical analysis are illustrated in Table 2, revealing that the classification accuracy of our algorithm exhibited significant differences compared to all baseline algorithms, with a p value of less than 0.05. This indicates a statistically meaningful advantage of our proposed algorithm over the alternatives.

**FIGURE 7:**
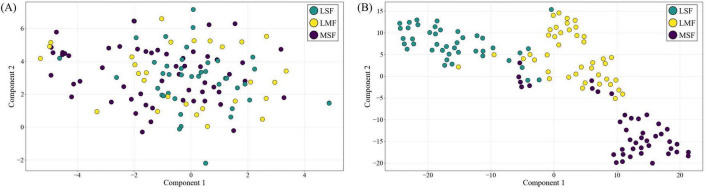
The t-SNE feature visualization of FN-SSIR. **(A)** High-dimensional input data projected into 2D space. **(B)** Corresponding feature representations from the FN-SSIR.

We visualized the high-dimensional features of subject S1 using t-SNE to compare the raw input data with the feature representations from the FN-SSIR. As shown in Figure 7, the model output (right) formed more compact clusters and clearer class boundaries compared to the input data (left), suggesting that the FN-SSIR effectively captured discriminative patterns for classification.

**FIGURE 8:**
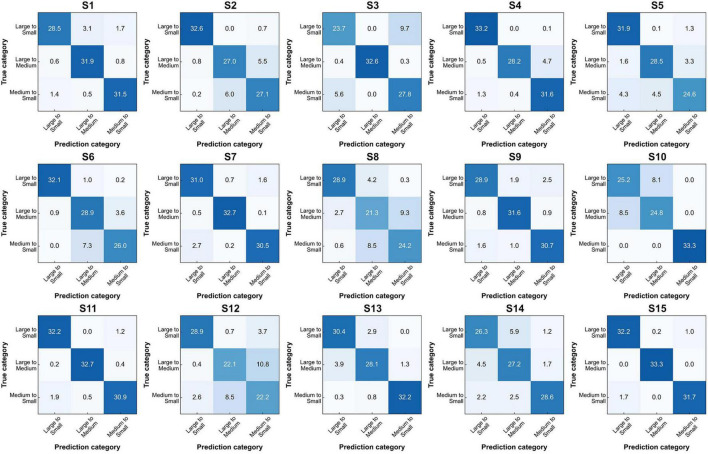
Confusion matrices for FN-SSIR classification across S1–S15 subjects.

To further illustrate the classification capability of our model, we plotted confusion matrices for all 15 subjects (Figure 8), providing a comprehensive overview of its performance across individual subjects. True positives and true negatives are evenly distributed, and the proportions of false negatives and false positives are relatively small. The results demonstrate that the proposed approach can decode mental imagery across various modalities without exhibiting bias toward particular tasks.

**FIGURE 9:**
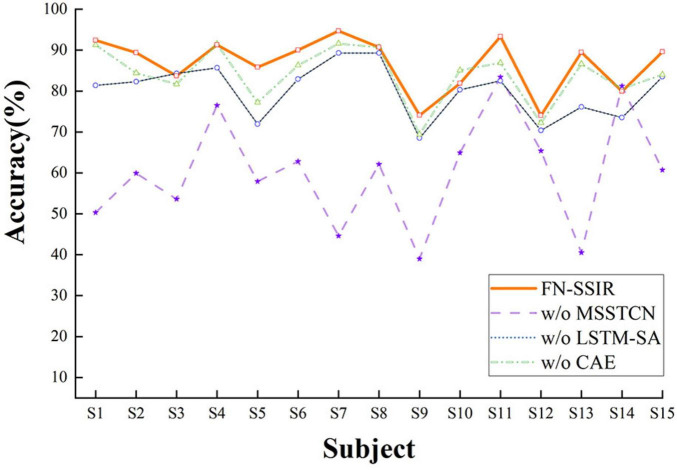
Ablation study results of the FN-SSIR. W/o, without the component; MSSTCN, multi-scale spatial-temporal convolutional module; CAE, convolutional auto-encoder module; LSTM-SA, LSTM with self-attention module.

In addition, we performed an ablation study to assess the contributions of the three key components in FN-SSIR: MSSTCN, CAE, and LSTM-SA. Each module was removed individually while keeping the remaining architecture intact. All experiments were conducted under identical conditions. As illustrated in Figure 9, removing any of the modules led to a noticeable decrease in accuracy, with the absence of the MSSTCN module causing the most significant performance drop. These results highlight the complementary roles of the three modules and underscore the importance of multi-scale temporal modeling.

**FIGURE 10:**
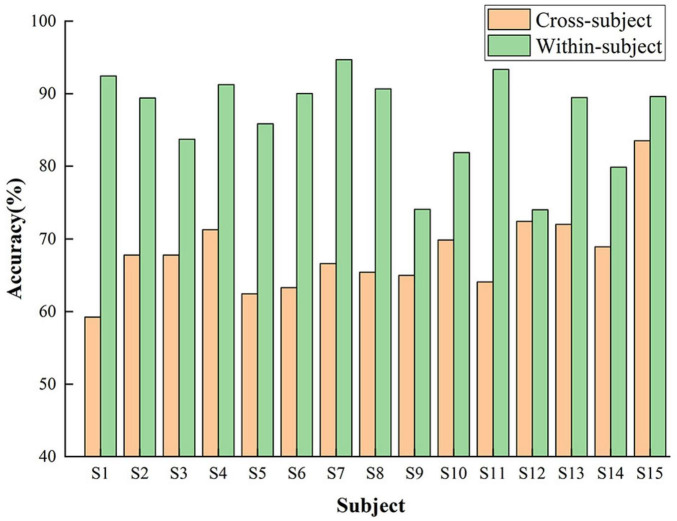
Cross-subject classification accuracy of the FN-SSIR using leave-one-out validation.

To evaluate the model’s generalization across subjects, we performed leave-one-subject-out cross-validation. In each iteration, one subject was held out as the test set, while the model was trained on the remaining 14. The classification accuracy for each subject is shown in Figure 10. The model demonstrated moderate to strong generalization, with consistent performance observed across most subjects. Notably, the highest accuracy was achieved on subject S15, while subject S1 exhibited comparatively lower performance, reflecting potential inter-subject variability in EEG patterns.

To further verify the generalizability of FN-SSIR, we additionally carried out comparative experiments on Dataset2 (a publicly available four-class MI-EEG dataset).

Following the official protocol of the BCI Competition IV-2a dataset, we used the first session of each subject for training and the second session for testing. The proposed model achieved an average classification accuracy of 78.4% with a standard deviation of 13.0%. To assess statistical significance, we conducted paired *t*-tests comparing FN-SSIR with each baseline method. The results showed in Table 3 that FN-SSIR significantly outperformed OVRCSP (*p* = 0.0099), 2D CNN-LSTM (*p* = 0.0002), EEGNet (*p* = 0.0020), MSTCN-AM (*p* = 0.0024), and BrainGridNet (p < 0.0001). No significant difference was found between FN-SSIR and FBMNet (*p* = 0.1652).

**TABLE 3:** Classification accuracy (%) comparison for different algorithms on dataset 2.

	FN-SSIR	BrainGird Net	FBMS Net	2d CNN- LSTM	OVR-CSP +SVM	EEGNet	MSTCN- AM
S1	88.5	64.6	87.8	77.4	80.2	85.8	82.6
S2	63.2	41.7	63.9	55.6	64.6	61.6	57.6
S3	92.4	71.2	88.9	85.4	86.1	88.5	85.4
S4	75.0	47.9	68.4	60.1	55.2	67.0	66.0
S5	64.6	47.6	72.2	51.7	47.9	55.9	60.4
S6	61.1	43.4	52.4	51.4	55.2	52.1	45.8
S7	94.8	53.8	86.5	78.1	77.8	89.6	80.6
S8	86.1	71.2	82.3	77.8	86.5	83.3	81.3
S9	80.2	71.2	79.9	78.8	75.3	79.5	80.9
Mean ± Std	78.4 ± 13.0	56.9 ± 12.6***	75.8 ± 12.5	68.5 ± 13.5**	69.9 ± 14.5**	73.7 ± 14.7**	71.2 ± 14.1**

Stars denote the statistically significant difference between two algorithms (where *denotes *p* < 0.05, ^**^denotes *p* < 0.01, and ^***^denotes *p* < 0.001).

This evaluation was mainly conducted to verify the generalizability of FN-SSIR beyond the experimental conditions of Dataset1.

## 5 Discussion

In current MI-BCI, the paradigms for imagining the force intensity of limb typically adopt the movements of hand grip. These paradigms are constantly concerned with static force under the right upper-limb, which belongs to the motor imagery of unilateral upper-limb. Nevertheless, it is hard to achieve interaction of dynamic force between robots and patients in brain-controlled rehabilitation robot systems. This limitation consequently restricts its application in rehabilitation training. To address this issue, recently researchers have introduced MI paradigms with force intensity that focuses on dynamic actions, such as wiping a tabletop and lifting a water bottle (Sheng et al., 2023; Karakullukcu et al., 2024). This development expands the category of MI with different force levels under unilateral upper-limb dynamic actions. However, the force of these paradigms is fixed in a single trial and it is difficult to meet the requirements of naturalistic interactions in brain-controlled rehabilitation robot systems.

Therefore, we design a MI paradigm in which subjects imagined the force intensity variations of dynamic movements in the unilateral upper-limb. Three combinations of variations in force intensity were introduced: large-to-medium, large-to-small, and medium-to-small. It increased the number of MI thinking categories with force intensity variations under a unilateral upper-limb dynamic state. Then, we analyze the EEG signals induced by this paradigm in the time, time-frequency, and spatial domain. It showed that there were significant ERD phenomena in both alpha and beta frequency bands, in which subjects imagined the force intensity variations of dynamic movements in a single trial. And the ERD phenomenon gradually weakened as the force intensity decreased. Meanwhile, brain topographic maps showed that the activation extent of the contralateral sensorimotor area decrease as the force intensity reduces. It displays a pattern of brain region responses from strong to weak. Although the EEG features overlap in the spatial domain when the subjects imagined variations of force intensity, they still exhibit distinct regional differences. We found individual differences in ERD phenomena across subjects by brain topographic maps segmented with time windows. Additionally, in the time-domain waveforms of the FC3, we can observe the significant differences in negative shift between the three classes of force intensity. These results establish a foundation for the development of our algorithm aimed at recognizing EEG features induced by our paradigm.

As a traditional extraction method of EEG features, OVR-CSP+SVM needs manual extraction of features, and the process of extraction is independent of the classification. CSP focuses on extracting linear spatial features of EEG signals, which makes it difficult to capture complex nonlinear relationships. Although SVM deals with nonlinear problems through kernel functions, its performance is limited under high-dimensional features. Both EEGNet and 2D CNN-LSTM use the end-to-end approach without manual extraction of features. They automatically extract features from raw EEG data and perform classification directly. However, fixed-size convolutional kernels or fixed time steps are used in these network models, resulting in their ability to extract features only from a single scale. This constraint hinders the ability to capture features of EEG signals across different frequency bands and time scales. Thus, it limits the classification performance.

FBMSNet employs mixed depthwise convolution and spatial filtering to efficiently extract multi-scale temporal-frequency-spatial features. MSTCN-AM combines an attention mechanism with a multi-scale convolutional network to extract fine-grained time-frequency and spatial features, with the attention module further reducing information redundancy. Although these models effectively capture multi-scale features in the time-frequency and spatial domains, they still rely on a one-dimensional spatial representation. BrainGridNet addresses this limitation by employing a two-branch depthwise CNN to process 3D EEG data, thereby enriching spatial information and optimizing computational efficiency. However, all the above methods focus primarily on recognizing ERD features in EEG signals, leading to limited feature diversity. In summary, above algorithms have some limitations in recognizing the features of EEG signals corresponding to MI tasks with different combinations of force intensity variations.

For the recognition of EEG features associated with MI involving force intensity variations, we propose a network algorithm (FN-SSIR). The spatial-temporal-enhanced features of EEG signals in multiple dimensions are extracted by the multi-scale spatial-temporal convolution module. This is achieved by integrating multi-scale convolutional kernels with three-dimensional convolutions, allowing the model to effectively learn spatial dependencies and temporal dynamics simultaneously. The use of small-scale kernels enables the capture of subtle, localized features from neighboring electrodes, while large-scale kernels aggregate broader contextual information across brain regions. This design balances the need for local sensitivity and global awareness. The convolutional auto-encoder module (CAE) progressively extracts the temporal information in the EEG signals through multiple layers of convolutions. It also utilizes the pooling layer to reduce dimensions, thereby mitigating noise and producing a compact representation of features, which helps to enrich the diversity of extracted patterns. Then, the decoder reconstructs the features through transposed convolution. To address the individual differences when subjects performing MI tasks, we introduce an LSTM module with a self-attention mechanism to dynamically focus on the input sequence. This module applies temporal weighting to the output features from the MSSTCN module and CAE module. By combining the long range temporal modeling capacity of LSTM with the selective focusing ability of attention, the network is better equipped to capture subtle temporal features associated with force intensity variations in motor imagery.

The classification results on Dataset1 (the MI-EEG dataset involving force intensity variations) are summarized in Table 2. Our proposed algorithm achieved a classification accuracy of 86.7% ± 6.6%, outperforming all baseline methods. Moreover, the accuracy differences between our algorithm and the baseline algorithms were statistically significant (*p* < 0.05). For Dataset2 (the public BCI Competition IV-2a dataset), FN-SSIR also demonstrated good generalizability, achieving competitive performance despite being originally designed for force intensity variations MI tasks. FN-SSIR not only achieves high average performance but also demonstrates statistically significant improvements over most baseline methods in Dataset2. The lack of significant difference between FN-SSIR and FBMNet may be attributed to their comparable ability to capture complex temporal-spatial patterns. However, FN-SSIR still offers better performance stability across subjects.

In this study, one limitation lies in the potential subjective differences in how individual subjects perceive force variations, which could affect the performance of the MI tasks. These individual differences may lead to inconsistent task performance across subjects. To address this, future work could incorporate additional modalities, such as electromyographic (EMG) signals or force-feedback devices, during the MI training phase to standardize the force variations and improve the control of the MI tasks. While the FN-SSIR captures spatiotemporal EEG features effectively, it can be further refined to better handle individual differences. Future work could explore adaptive feature extraction or transfer learning to enhance the model’s generalization and classification performance across subjects.

## 6 Conclusion

For the limitations of current MI-BCI systems in force control of rehabilitation robots, we designed a MI paradigm for force intensity variations under dynamic movements of the unilateral upper-limb. The category of thinking about MI tasks with force intensity is enriched by the introduction of different combinations of force intensity variations. Based on this, the FN-SSIR is proposed, combining multi-scale spatial-temporal convolution with spatial-temporal-enhanced strategy, a convolutional auto-encoder for information reconstruction, and LSTM with self-attention to extract and classify multidimensional features. For our MI-EEG dataset involving force intensity variations, FN-SSIR achieved an average classification accuracy of 86.7% and the lowest standard deviation of 6.6%, outperforming all baseline methods. These results further validate the effectiveness and robustness of the proposed algorithm. In addition, evaluations on the public dataset suggested that FN-SSIR exhibits a certain degree of generalizability. By effectively capturing and identifying the subtle variations that occur during MI with varying force intensity, our approach supports more natural interaction in future MI-BCI rehabilitation robots.

## Data Availability

The original data and materials presented in this article can be obtained from the corresponding authors upon request.
